# A new perspective on Alzheimer’s disease: m6A modification

**DOI:** 10.3389/fgene.2023.1166831

**Published:** 2023-05-09

**Authors:** Lei Xia, Fan Zhang, Yulu Li, Yuemi Mo, Lingqiu Zhang, Qianhua Li, Minghuang Luo, Xiaotao Hou, Zhengcai Du, Jiagang Deng, Erwei Hao

**Affiliations:** ^1^ Guangxi Scientific Experimental Center of Traditional Chinese Medicine, Guangxi University of Chinese Medicine, Nanning, China; ^2^ Guangxi Key Laboratory of Efficacy Study on Chinese Materia Medica, Nanning, China; ^3^ Guangxi Collaborative Innovation Center of Study on Functional Ingredients of Agricultural Residues, Nanning, China; ^4^ Guangxi International Zhang Medicine Hospital Affiliated to Gungxi University of Chinese Medicine, Nanning, China; ^5^ Guangxi Key Laboratory of TCM Formulas Theory and Transformation for Damp Diseases, Guangxi University of Chinese Medicine, Nanning, China

**Keywords:** Alzheimer’s disease, m6A modification, synaptic loss, animal model, techniques

## Abstract

As a neurodegenerative disease, Alzheimer’s disease (AD) is characterized by synaptic loss, extracellular plaques of amyloid accumulation, hyperphosphorylation of tau, and neuroinflammation. Various biological processes are affected by epitranscriptomic modifications, which regulate the metabolism of mRNA in cells and regulate the expression of genes. In response to changes in m6A modification levels, the nervous system becomes dysfunctional and plays a significant role in the development of Alzheimer’s disease. As a result of recent research, this paper reviews advances in the understanding of the regulatory mechanisms of m6A modification in the occurrence and development of AD. In addition, the article discusses recent research techniques related to animal models of m6A and AD. Furthermore, it discusses the possibility of studying the pathogenesis of AD at the level of the epitranscriptome, identifying early diagnostic markers, and screening for effective treatment options.

## 1 Introduction

AD is one of the leading causes of dementia and a growing global health problem. Moreover, the vast majority of AD patients are elderly. With the aging of the population, the increasing prevalence of AD is becoming one of the major challenges for the social public health system. Although the understanding of the pathogenesis of AD has made great progress in the past few decades, there is still no cure for AD. The key to initiating treatment is an early and accurate diagnosis. However, preclinical AD is difficult to detect, and patients do not have functional impairments in daily activities, although the cerebral cortex and hippocampus have pathological changes (Dubois et al., 2016). Presently, AD is primarily diagnosed through positron emission tomography (PET) and analysis of cerebrospinal fluid (CSF) proteins. Even though it has a high level of accuracy, AD has already progressed to a chronic stage when pathological symptoms are observed. In light of this, biomarkers may be able to assist in the early detection of Alzheimer’s disease.

m6A is a ubiquitous internal chemical modification in mRNA, which regulates mRNA expression, splicing, decay and translation, and is involved in cell development, embryonic development and stress response (Zhao et al., 2017). The distribution and motif of m6A during human development vary across tissues and developmental processes (Zhang et al., 2020). m6A modification sites are biased to the Coding sequence (CDS) and 3′Untranslated Region (3′UTR) regions, and even a single modification site has a large effect on function (Zhang et al., 2020). A number of recent studies have shown that m6A is abnormally expressed in the brain of AD patients. It may be possible to study the pathogenesis of AD and find new biomarkers from the perspective of m6A modification. Therefore, this article discusses the role of m6A modifying enzymes in the pathological process of AD, and summarizes the animal models used for AD research and the new research techniques related to m6A.

## 2 Pathogenesis, diagnosis and treatment of AD

### 2.1 Pathogenesis of Alzheimer’s disease

**FIGURE 1:**
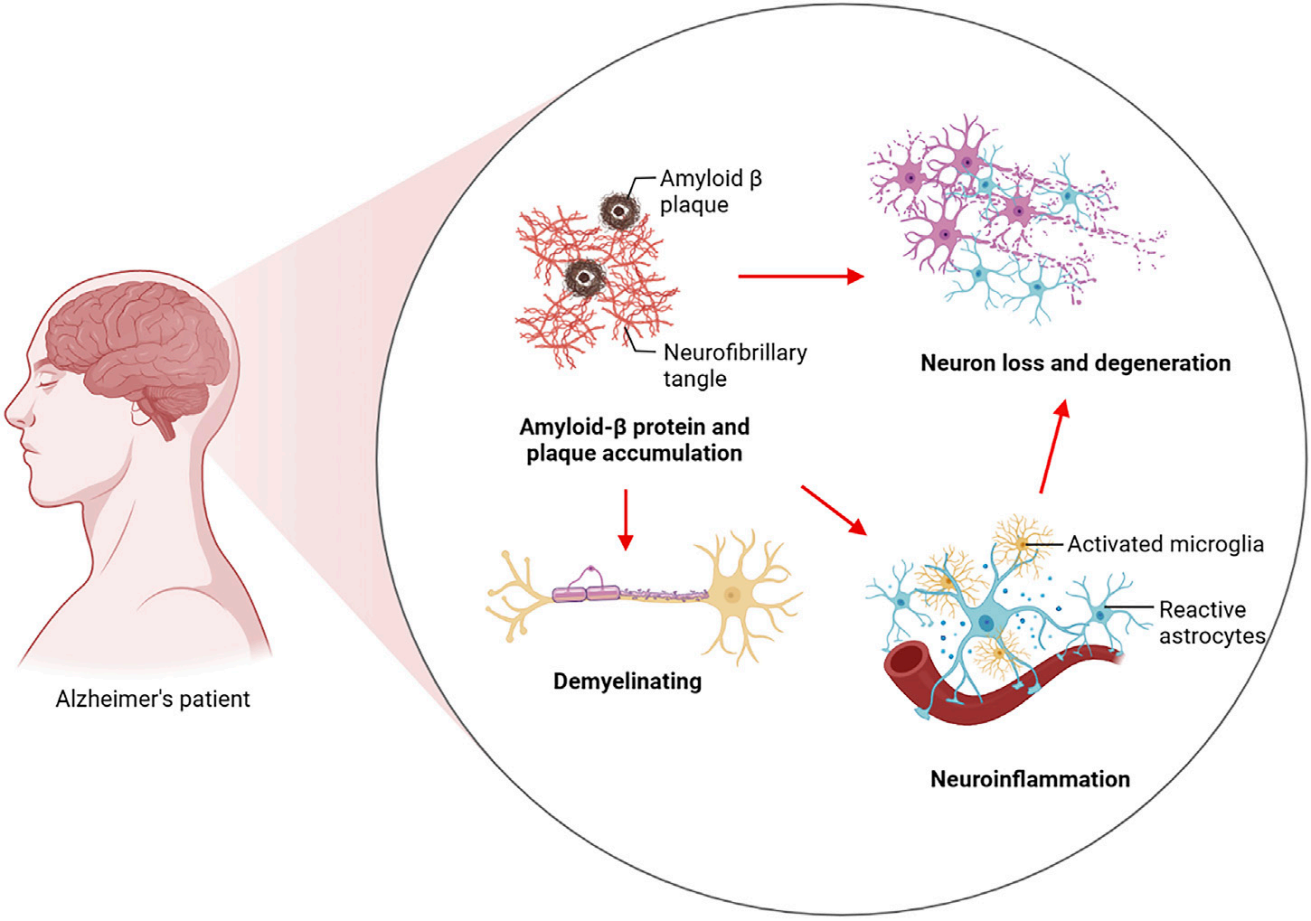
Pathogenesis of Alzheimer’s disease. Aβ induces AD by destroying neuronal cells and induces tau hyperphosphorylation to drive the development of tau pathology to form NFTs and then lead to the harmful cascade of neurodegeneration. These include Aβ-induced failure of OPCs to differentiate into oligodendrocytes, resulting in white matter abnormalities and ultimately demyelination of nerve cells. Aβ disrupts synaptic plasticity and activates microglia to promote inflammation, ultimately leading to synaptic loss and neuroinflammation.

AD is a chronic neurodegenerative disease. As is evident from clinical observations, AD usually typically manifests itself as a progressive decline in higher cognitive functions as well as memory loss. The preclinical stage of AD can be sustained for a long period of time. An initial pathological examination of the cerebral cortex and hippocampus revealed that the patient was experiencing mild memory loss. This is followed by memory loss, emotion changes, dyslexia and, in later stages, abnormal neuropsychiatric symptoms (Knopman et al., 2021; Yun-Ling Zhang et al., 2021). As a pathological condition, AD is characterized by widespread loss of neurons in the brain and the formation of two characteristic proteins, amyloid plaques and neurofibrillary tangles (NFTs), which are produced by hyperphosphorylation of tau. As a result, there is a loss of synaptic plasticity, demyelination, neuroinflammation, and neurotoxicity in the AD brain. In terms of the pathogenesis of AD, two major theories have been proposed: a cascade involving amyloid *ß* (Aβ) and hyperphosphorylation of tau protein. Aβ deposits as neuroinflammatory plaques and induces AD through its destruction of neuronal cells. Tau hyperphosphorylation then leads to tau pathology leading to NFTs and subsequently to the harmful cascade of neurodegeneration (Busche and Hyman, 2020; Zhang et al., 2021). At the same time, Aβ is considered to be an important factor for neuronal and synaptic dysfunction in the progression of AD. This substance disrupts synaptic plasticity, inhibits long-term potentiation (LTP) in the hippocampus, and causes oxidative stress in the brain through the production of reactive oxygen species (Lustbader et al., 2004). Increasing oxidative stress can lead to oligodendrocyte death and dysfunction, resulting in the failure of Oligodendrocyte progenitor cells (OPCs) to differentiate into oligodendrocytes, which eventually results in abnormalities in the white matter and demyelination of nerves (Nasrabady et al., 2018). There are microglia which are macrophages in the brain, and microglia have a role to play in the removal of amyloid beta. However, with the massive accumulation of Aβ precipitates, in turn, Aβ activated microglia produce inflammatory factors, which in turn cause neuronal cell damage and eventually lead to neuroinflammation (Hickman et al., 2008; Babcock et al., 2015). There was an immune response observed in mice transgenic for human APP (hAPP) at hippocampal and cortical synapses that were engulfed by microglia prior to plaque deposition (Shi Q. et al., 2017) (Figure 1).

### 2.2 Diagnosis and treatment of Alzheimer’s disease

The long latency period in the early stages of AD and the lack of obvious clinical manifestations lead to difficulties in the diagnosis of AD. Amyloid accumulation (A), hyperphosphorylation of tau (T) and neurodegeneration (N) are considered to be biomarkers of AD (Jack et al., 2018). Nevertheless, there are operational limitations associated with the use of ATN for the diagnosis of AD in clinical settings. Clinicians still lack effective and efficient biomarkers and detection methods. In terms of drug treatment, only five drugs approved by the FDA for the treatment of AD are on the market, and these drugs are only effective for the treatment of AD symptoms (Briggs et al., 2016). A number of new drugs have been developed for AD in recent years, but none have reached the market. Because AD pathology is diverse and the brain is complex, treating AD is challenging.

In the prevention, diagnosis and treatment of disease, biomarkers play a crucial role. Detecting effective and characteristic markers of AD is important for the prevention and diagnosis of the disease, especially at an early stage. Transcriptomics of epitranscriptomic changes is an emerging field of biological research. Unlike epitranscriptomic modifications of DNA and histones, which affect the expression of genes, chemical modifications of RNA are thought to play a significant role in the occurrence and development of diseases (Fu et al., 2014). Methylation is one of the most prominent forms of RNA modification. Currently, m6A (N6-methyladenosine), which has the highest abundance of internal modifications in messenger RNA (mRNA), is the subject of the most intensive investigation (Zhao et al., 2017). The methylation modifications of specific genes by m6A can serve as potential biomarkers and therapeutic targets in oncology and cardiovascular disease research (Sun et al., 2019; Wu et al., 2020). α-ketoglutarate-dependent dioxygenase alkB homologue 5 (ALKBH5) can reduce the m6A modification of NANOG mRNA, which plays an imperative role in the maintenance of cancer stem cells, thereby enhancing its stability in breast cancer (Zhang et al., 2016a; Zhang et al., 2016b). There is a significant regulatory role played by fat mass and obesity-associated protein (FTO) in the development of heart failure. By selectively demethylating myocardial contractile transcripts, FTO can increase the expression of these transcripts and prevent their degradation, thus protecting the contractile function of cardiomyocytes (Mathiyalagan et al., 2019). The role of m6A modification in the nervous system has attracted considerable attention in recent years. The level of m6A modification in the brain increases with brain development and exhibits tissue specificity, strongly suggesting that m6A is closely related to brain function (Meyer et al., 2012). Meanwhile, the level of m6A in the brains of AD patients differs from that of normal brains, attracting the attention of researchers (Shafik et al., 2021). The discovery of m6A may prove to be a new breakthrough in the search for biomarkers of AD.

## 3 m6A methylation modification

**FIGURE 2:**
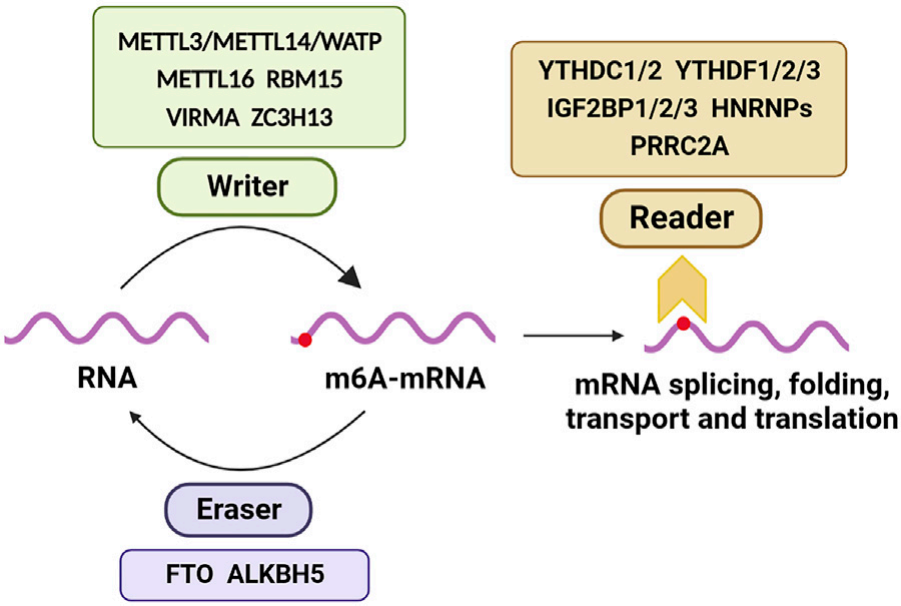
m6A methylation modification process.

Approximately 80% of RNA methylation modifications in eukaryotes occur through m6A methylation. A m6A modification enzyme system consists of a methylation transferase system (Writers), a demethylase system (Erasers), and a binding protein system (Readers). As a result of their ability to add, remove or identify m6A modification sites, these regulators are able to alter critical biological processes such as splicing, processing, RNA stability, translation, and cellular metabolism. M6A modification plays a significant role in brain development and function as a post-transcriptional regulator. As neurons mature, m6A’s expression levels increases, and m6A-seq functional classification analysis reveals that most of m6A’s target genes are involved in neural development, synaptic transmission, and postsynaptic function (Chang et al., 2017; Huang et al., 2020). The methyltransferase-like protein 3 (METTL3), also known as Writer, is a binding protein for S-adenosylmethionine. METTL3 is a binding protein of S-adenosyl methionine (SAM) that acts as the core of the methyltransferase complex and is involved in methylating mRNA (Zeng et al., 2020). METTL14, another component of the methyltransferase complex, co-localizes with METTL3 in the nucleus and forms a stable heterodimer that enhances METTL3’s catalytic activity (Wang et al., 2016). Wilms tumor 1-associated protein (WTAP) regulates the recruitment of m6A methyltransferase complexes to mRNA targets (Ping et al., 2014). It was discovered that m6A modification is dynamic and reversible when the demethylase fat mass and FTO was discovered (Jia et al., 2011). ALKBH5, which is a demethylase like FTO, belongs to the α-ketoglutarate-dependent dioxygenase protein family and relies on α-ketoglutarate/Fe2+ to perform demethylation function (Zheng et al., 2013). m6A-binding proteins are mainly involved in recognizing m6A-modified mRNA chains and are involved in the splicing, folding, transport, and translation of mRNA. The role of m6A methylation in the development of diseases, including neurological disorders, has attracted increasing attention in recent years. According to the brain synaptic tagging hypothesis, ALKBH5-mediated m6A demethylation can alter RNA methylation and result in synaptic dysfunction as well as reversible disease conditions (Martinez De La Cruz et al., 2021) (Figure 2; Table 1).

**TABLE 1:** m6A Modifying Enzymes.

Type	Regulator	Function	References
Writer	METTL3	As the core of the methyltransferase complex, it catalyzes the m6A modification of adenylate on mRNA	Zeng et al. (2020)
	METTL14	Forms a stable heterodimer with METTL3 to enhance the catalytic effect of METTL3	Wang et al. (2016)
	WTAP	Promotes the localization of methyltransferase complexes to nuclear plaques	Ping et al. (2014)
	METTL16	U6 spliceosome small nuclear RNA methyltransferase, is required for the maintenance of SAM homeostasis	Pendleton et al. (2017)
	RBM15	Localized in the nucleus and may bind to RNA by interacting with the spliceosome	Patil et al. (2016)
	VIRMA	Mediate preferential methylation of mRNA 3′UTR and near stop codons	Yue et al. (2018)
	ZC3H13	Immobilizes a complex containing WTAP, Virilizer, and Hakai in the nucleus	Wen et al. (2018)
Eraser	FTO	Demethylation of m6A residues of targeted transcripts	Wei et al. (2018)
	ALKBH5	Affects mRNA export and RNA metabolism through demethylation	Zheng et al. (2013)
Reader	YTHDF1	Promotes cap-independent translational regulation of m6A modified RNA transcripts in the 5′UTR region	Wang et al. (2015)
	YTHDF2	Recognizes m6A modifications and reduces the stability of target transcripts	Wang et al. (2015)
	YTHDF3	Cooperates with YTHDF1 to promote protein synthesis	Shi et al. (2017a)
	YTHDC1	Affects mRNA alternative splicing and nuclear export	Xiao et al. (2016)
	YTHDC2	Improve translation efficiency	Hsu et al. (2017)
	HNRNPA2B1	Affects the processing and alternative splicing of primary microRNA	Alarcon et al. (2015)
	HNRNPC	Binding with transcripts and regulates mRNA abundance and splicing	Liu et al. (2015)
	HNRNPG	Binding with transcripts and regulates mRNA abundance and splicing	Liu et al. (2017)
	IGF2BPs	Cooperates with YTHDF1 to promote protein synthesis	Huang et al. (2018)
	PRRC2A	Binding to target mRNA promotes m6A modification	Wu et al. (2019)

## 4 m6A methylation in Alzheimer's disease

### 4.1 The role of m6A in the development of Alzheimer’s disease

AD is a neurodegenerative disease associated with aging and is the leading cause and major risk factor for dementia in the elderly (Hou et al., 2019). There is an association between m6A modification and neural development (Nussbaum and Ellis, 2003). There was a decrease in m6A methylation levels in the brain of AD patients compared to cognitively intact older adults (Castro-Hernandez et al., 2023). The m6A methylation is often enhanced near the stop codon and in the 3′UTR to affect mRNA expression. A 5XFAD mouse model exhibits lower levels of m6A methylation than a wild type mouse, and the 3′UTR of wild type mice is significantly enriched in m6A modification (Shafik et al., 2021). It is possible that the abnormal levels of m6A in the brains of AD patients and AD model mice are related to the pathological changes that occur in AD brains.

### 4.2 The role of m6A on cognitive impairment in the pathogenesis of AD

During the development of the human brain, the degree of methylation of m6A changes dynamically, and its expression is spatially specific at specific times (Shafik et al., 2021). There is a reduction in RNA m6A modification in large pyramidal neurons of AD patients compared to that of normal elderly individuals. This is associated with pathological findings including reduced synapses, neuronal atrophy, and increased gliosis. A significant proportion of neurons in the brains of Alzheimer’s patients have been lost. It has been demonstrated that the brain’s hippocampal neurons are closely related to memory, and that damage to these neurons will lead to memory impairment and the manifestations of dementia (Ginsberg et al., 2012). Generally speaking, mild cognitive impairment (MCI) is considered a stage in the progression of more severe dementia and a precursor to AD.

Aβ is considered to be an important factor in neuronal and synaptic dysfunction in the progression of AD, which can disrupt synaptic plasticity, LTP in the hippocampus, and induce the production of reactive oxygen species (Lustbader et al., 2004). Synaptic loss causes cognitive decline, and this alteration in synaptic function is thought to be the primary mechanism underlying AD disorders (Henstridge et al., 2016). There is evidence that m6A is involved in memory formation and contributes to synaptic plasticity (Zhao et al., 2021). In AD patients, the hippocampus expressed a decreased level of the m6A methylases METTL3 and MELLT14 as well as WTAP. Several important synaptic genes, including α-amino-3-hydroxy-5-methyl-4-isoxazole-propionic acid (AMPA) and N-methyl-d-aspartic acid receptor (NMDA), were hypermethylated and their expression was significantly reduced in AD mice’s brains compared with control mice’s brains (Han et al., 2020). The middle temporal gyrus is the first area of the brain to show symptoms of AD. The expression of METTL3 is significantly decreased in the middle temporal gyrus of MCI patients, while the expression of METTL14 and FTO is unaffected (Zhao et al., 2021)c. METTL3 has an important in maintaining neuronal survival and synaptic structure, it has a structural and functional role in modifying neural circuits at the synaptic level. Aβ oligomers inhibited METTL3 expression and promoted the reduction of postsynaptic density protein 95 (PSD95) (Zhao et al., 2021). On the contrary, overexpression of METTL3 *in vivo* rescues synaptic damage and cognitive deficits induced by AβO (Zhao et al., 2021). Accordingly, *Mettl3* knockout mice in the hippocampus displayed memory loss and increased oxidative stress, resulting in DNA damage and activation of Caspase 3, a critical factor in neuronal programmed cell death. In addition, postsynaptic deficits and neural mutations result from the knockdown of *Mettl3* (Zhao et al., 2021). In other words, METTL3 contributes to the consolidation of hippocampal-dependent memory by promoting the translation of neuronal early response genes, thereby promoting the consolidation of long-term memory (Zhang et al., 2018). It can be seen that METTL3 is an important target of Aβ-induced synaptic loss in the hippocampus of AD brain, and the down-regulation of METTL3 causes a series of reactions that still need to be further studied. This also provides the possibility of METTL3 as a potential biomarker for AD.

In addition to METTL3, other m6A modifying enzymes also play a role in brain memory formation. In the case of Mettl3 and Mettl14 deficient mice, synaptic plasticity, memory, and learning are impaired (Koranda et al., 2018; Zhang et al., 2018). By contrast, *Fto* knockout mice increased brain m6A abundance and improved memory consolidation. The expression of Fto is reduced and the methylation of mRNA increases in response to fear conditions. Specifically, Fto may be responsible for repressing pre-memory transcript translation as well as promoting the translation of pre-memory transcripts. In response to neuronal stimulation, YTHDF1 promotes the protein synthesis of target transcripts in the hippocampus of adult mice, thereby facilitating learning and memory. It has been established that Ythdf1 knockdown causes hippocampal degeneration as well as deficits in memory and learning, whereas supplementation with Ythdf1 resolves behavioral and synaptic deficits (Shi et al., 2018). Thus, Ythdf1 promotes long-term changes in synaptic plasticity, which facilitate memory formation (Shi et al., 2018).

### 4.3 The role of m6A on demyelination in the pathogenesis of AD

In the central nervous system, oligodendrocytes, a subclass of glial cells, play an important role in myelination, which constitutes approximately 50% of brain cells (Kuhn et al., 2019). Aβ-induced oxidative stress can lead to oligodendrocyte death and dysfunction, resulting in white matter abnormalities (Nasrabady et al., 2018). Studies of neuroimaging in AD have demonstrated that significant white matter damage and myelin loss occur during the early stages of the disease, often before Aβ plaques and NETs become more apparent (Nasrabady et al., 2018). Olig2 and NG2 are often expressed in oligodendrocytes as Aβ plaque-associated proteins. Aβ plaque-associated protein induces OPCs senescence, turning them into senescent cells with a pro-inflammatory phenotype. In this condition, oligodendrocytes may not differentiate into myelin oligodendrocytes, which may contribute to cognitive deficits and neuroinflammation in AD (Zhang et al., 2019). Recent studies have indicated that Proline rich coiled-coil 2 A (PRRC2A), a novel m6A reader, is highly expressed in oligodendrocytes and regulates the expression of Olig2 mRNA by interacting with PRRC2A, the loss of PRRC2A results in demyelination (Wu et al., 2019). As opposed to this, Fto-mediated m6A demethylation promotes the degradation of Olig2 mRNA and contributes to the reduction of myelin in mice. Interestingly, PRRC2A is characterized by a novel domain that binds methylated RNA specifically and competes with YTHDF2 for RNA binding (Wu et al., 2019). The pathophysiology of AD is largely influenced by myelination (Mathys et al., 2019). As well, METTL14 is essential for the maturation of oligodendrocytes and the formation of myelin in the central nervous system, and the splicing of neural bundle protein mRNA in oligodendrocytes is affected by m6A methylation modifications (Xu et al., 2020).

### 4.4 The role of m6A on neuroinflammation in the pathogenesis of AD

Inflammatory factors can be a double-edged sword. In the pathogenesis of AD, inflammatory factors can remove the deposited Aβ, but Aβ activates inflammatory factors produced by microglia and astrocytes, which guide more inflammatory reactions at the site of injury and further produce neuroinflammation (Dhapola et al., 2021). As part of the pathological development of AD, neuroinflammation can also occur, including the activation of inflammatory factors and the activation of astrocytes and microglia to perform immune functions. Numerous neurodegenerative diseases are characterized by microglia-mediated cellular inflammation. Among the many functions that microglia play in neural development, these include forming synapse connections, eliminating synapse connections, and phagocytosing cells. Microglia that have been activated can become M1 or M2 phenotypes. As M1 is primarily involved in inflammatory factors and neurotoxicity, and M2 is primarily involved in tissue repair and restoration of homeostasis, microglia in AD can be continuously activated to the M1 phenotype, resulting in inflammatory responses that ultimately result in irreversible neuronal damage (Tang and Le, 2016). Inducing different phenotypes of microglia allowed us to examine m6A modifications on mRNA and IncRNA, which indicated that RNA m6A methylation differs greatly among different microglia phenotypes. A significant increase in m6A modified genes was observed when microglia switched from M0 to M1 phenotype, and these genes may be involved in the inflammatory response (Li et al., 2021).

The activation of microglia in the brain of AD patients was correlated with neuroinflammation and increased levels of MHC-II and CD68 positive cells (Hopperton et al., 2018). Microglial cells that are unable to clear Aβ deposits begin to fail, with lipofuscin deposition and a “dystrophic” state of cell fragmentation from multiple cytoplasmic protrusions to spherical swelling, cytoplasmic fragmentation, and vesicle formation (Streit et al., 2020). A significant change in the methylation levels of multiple inflammation-related factors occurs during the activation of microglia into pro-inflammatory microglia (Li et al., 2021). Microglial inflammation induced by lipopolysaccharide was shown to be accompanied by increased METTL3 expression, and METTL3 overexpression was found to activate TRAF6/NF-κB to promote microglial inflammatory responses in an *in vitro* model (Wen et al., 2022). Furthermore, IGF2BP1 could regulate the activation of microglia in this model, and its expression levels increased significantly based on the LPS concentration (Ding et al., 2022).

### 4.5 The role of m6A and neurotoxicity in the pathogenesis of AD

The NETs is composed of filamentous aggregates of the microtubule-associated protein tau. During neurodegeneration, Tau protein undergoes phosphorylation modifications and conformation changes, which lead to its separation from microtubules and binding to aggregates of varying sizes. One of the causes of AD is believed to be these aggregates that are capable of producing neurotoxicity (Niewiadomska et al., 2021).

FTO expression was increased in the brain tissue of 3×Tg mice. As *Fto* is knocked down in neurons, tau protein is phosphorylated at a lower level, whereas FTO overexpression in neurons increases tau protein phosphorylation. By activating mTOR and its downstream signaling pathways, FTO induces Tau phosphorylation and the mTORC1 inhibitor rapamycin blocks this action, indicating that FTO induces Tau phosphorylation (p-Tau) based on mTOR signaling (Li et al., 2018). It has been demonstrated that HNRNPA2B1 functions as a m6A reader in the nucleus and that Tau oligomerization induces its translocation to the cytoplasm. It is interesting to note that HNRNPA2B1 promotes tau oligomerization but not pTau oligomerization. HNRNPA2B1 and pTau were able to interact more effectively with one another due to tau oligomerization facilitated by pTau. For tau oligomers to bind to m6A, HNRNPA2B1 is required. In the context of Tau oligomerization, METTL3 knockdown decreased m6A methylation and prevented HNRNPA2B1 accumulation in the cytoplasm. Knocking down HNRNPA2B1 may partially reverse neurotoxicity (Jiang et al., 2021). NOP2/Sun RNA methyltransferase 2 (NSun2) is well known as an m5C RNA methylase (Hussain et al., 2013). However, NSun2 also appears to function as an m6A RNA methylase for m6A modification of microRNA. NSun2 is located in the nucleus of neurons in the hippocampus and prefrontal cortex of the brain, and its expression is reduced in AD patients. Its downregulation promotes Tau phosphorylation (Kim et al., 2022). In contrast, NSun2 overexpression resulted in a significant reduction in phosphorylated tau levels, restored neuronal cell viability and reduced AβO-induced toxicity, indicating a neuroprotective role of NSun2. It is noteworthy that regions of the brain that were not affected by AD pathology, such as the cerebellum, had NSun2 levels comparable to those in the control cerebellum, indicating that NSun2 deficiency is limited to those brain regions most vulnerable to AD (Kim et al., 2022). Further, METTL3, also a ‘Writer’, can improve AD through the enhancement of autophagy clearance of p-Tau through a m6A-dependent STUB1 pathway (Tang et al., 2023).

### 4.6 The relationship between apolipoprotein E and m6A in AD

Most of the components of the brain are composed of lipids, and lipid metabolism is often associated with pathological conditions of the brain. APOE is a class of glycoproteins that are widely expressed in astrocytes, microglia, and vascular wall cells in the central nervous system (CNS). In addition to being involved in lipid transport and metabolism, APOE4 is one of the most prevalent genetic risk factors for late-onset Alzheimer’s disease (Chew et al., 2020). It has been shown in AD that APOE4 enhances microglia-induced inflammation, promotes neurodegeneration, alters lipid raft structure within neuroglia membranes, and accelerates the destruction of Aβ and tau proteins. According to the bioinformatics data, APOEɛ4 may have a close relationship to the m6A methylation regulators (METTL3, METTL16, YTHDC2, LRPPRC) in brain tissue from AD patients, but the exact mechanism needs to be determined by further experimental studies (Du et al., 2021).

**FIGURE 3:**
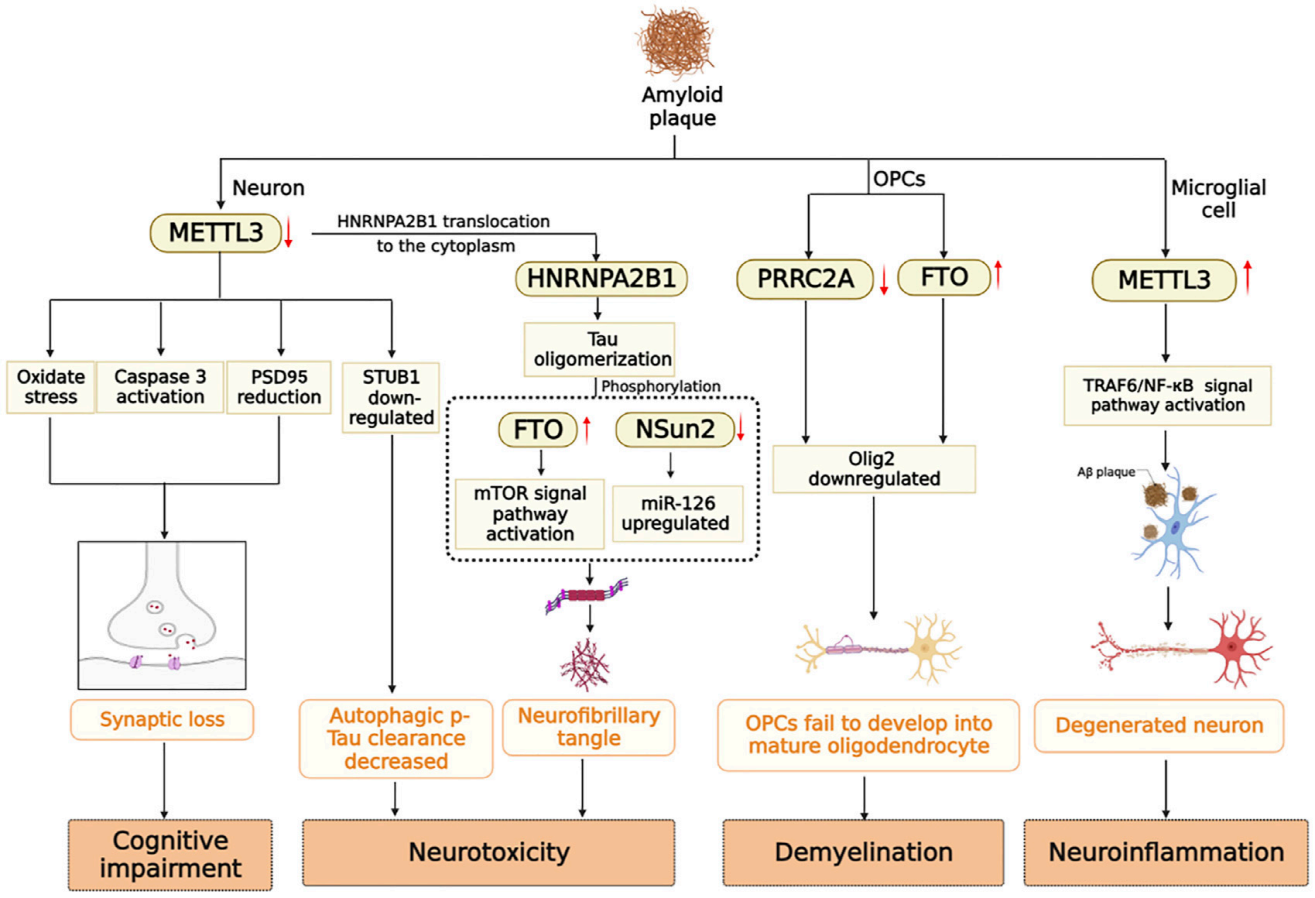
m6A modifying enzymes are involved in the pathogenesis induced by Aβ and Tau protein in AD brain.

In summary, cognitive impairment, white matter demyelination, neuroinflammation, and neurotoxicity are all associated with the presence of Aβ-insoluble starch and hyperphosphorylated Tau. Under stimulation of Aβ, the expression level of METTL3 in neurons is down-regulated, oxidative stress occurs, the apoptosis signaling pathway is activated, and PSD95 protein is reduced, which eventually leads to synaptic loss (Zhao et al., 2021). However, METTL3 expression is up-regulated in microglia, which activates the TRAF6/NF-κB signaling pathway and contributes to neuroinflammation (Wen et al., 2022). In addition, m6A modifying enzymes participate in the processes of Tau oligomerization, phosphorylation, and myelination. Multiple factors contribute to the pathogenesis of Alzheimer’s disease. Previously, it was mentioned that Aβ and Tau proteins promote each other. Down-regulation of METTL3 by Aβ stimulating in neurons leads to the translocation of HNRNPA2B1 originally located in the nucleus to the cytoplasm to promote tau oligomerization (Jiang et al., 2021). A notable observation was that METTL3 is accumulated in insoluble plaques in the *postmortem* brains of AD patients, and its expression level is positively correlated with Tau levels. In contrast, METTL3 expression was reduced in the hippocampal region (Huang et al., 2020). Aside from the modifying enzymes mentioned above, RBM15B, ALKBH5 and ELVAL3 showed significant changes in the m6A gene in patients with AD (Huang et al., 2020). Currently, there are limited studies on the signaling pathways involved in AD that are mediated by m6A-modifying enzymes. The study of m6A-modifying enzymes may provide insight into the occurrence and development of AD, as well as identify potential biomarkers and therapeutic targets for early detection of the disease (Figure 3).

## 5 AD animal model and related m6A studies

**FIGURE 4:**
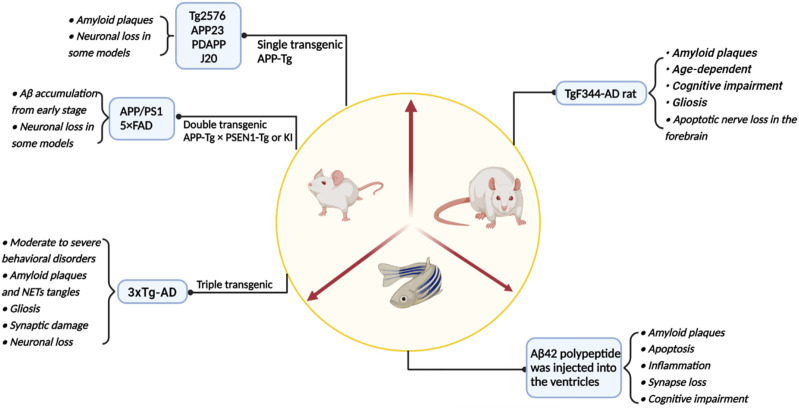
A summary of the main model animals used to construct AD models and their pathological representations.

Since the 21st century, the incidence of AD has increased year by year while it has also been extensively studied. To explore the pathogenesis of AD, researchers have not only studied brain samples, brain cell lines and genomes from *postmortem* AD patients and normal individuals. The studies of AD pathogenesis and the development of targeted drugs have also been significantly aided by a variety of animal disease models (Figure 4; Table 2).

**TABLE 2:** Role of m6A modifying enzymes in model animals and AD pathology.

Samples	Expression of m6A	Pathological features of AD	Effect	References
Human brain tissue	Expression of METTL3, METTL14, WTAP, FTO and YTHDF1 is decreased in the AD brain. METTL3 expression is significantly reduced in the middle temporal gyrus in AD.	Cognitive deficits, demyelination, neuroinflammation and neurotoxicity	RNA m6A modification was significantly reduced in large pyramidal neurons of the AD brain, but increased in microglia and astrocytes	Huang et al. (2020)
	NSun2 is located in the nucleus of neurons in the hippocampus and prefrontal cortex of the brain, and its expression is reduced in AD patients	Tau pathology	NSun2 downregulation promotes Tau phosphorylation	Kim et al. (2022)
shMettl3-injected mice	Level of m6A was reduced in neurons; Endogenous METTL3 protein was reduced in neurons	Cognitive deficits; Neuronal loss in the hippocampus; Dendritic spines and mushroom spines decreased; Increased activation of microglia	Activation of Caspase 9/Caspase 3 signal pathway; Increased oxidative stress and aberrant cell cycle events	Zhao et al. (2021)
Rat primary cortical neurons cell by AβO treatment	Expression of METTL3 was significantly decreased		Significant reduction of PSD95	Zhao et al. (2021)
AAV-METTL3 mice treated by AβO	METTL3 overexpression	Synaptic loss	AβO-induced synaptic loss was prevented, and cognitive and motor behaviors were normal	Zhao et al. (2021)
Mouse primary microglia cell treated by LPS	METTL3 expression was upregulated		Upregulation of TRAF6, NF-κB and inflammatory factor like IL-1β, IL-18, IL-6 and TNF-α; Overexpression of METTL3 promotes LPS-mediated inflammation in microglia by activated TRAF6/NF-κB pathway in an m5A-dependent manner	Wen et al. (2022)
SH-SY5Y and HT22 cells treated by A*β*_1-42_	Expression of METTL3 was decreased	The level of p-Tau was significantly increased	METTL3 enhanced autophagic p-Tau clearance through the m6A- IGF2BP1-dependent regulation of STUB1 mRNA	Tang et al. (2023)
APP/PS1 mice	Expression of METTL3 was decreased	The level of p-Tau was significantly increased	Overexpression of METTL3 can ameliorate hippocampal damage and Aβ deposition, and improve AD by enhancing p-Tau autophagy	Tang et al. (2023)
*Mettl3* ^*flox/flox*^ mice (Forebrain excitatory neuron-specific *Mettl3* conditional knockout mice)	Expression of METTL3 was decreased		METTL3-mediated long-term memory formation is dependent on its m6A methyltransferase function	Zhang et al. (2018)
*Mettl3*-KO zebrafish	Expression of METTL3 was decreased		Defects in embryos	Ping et al. (2014)
*Tyrobp* ^*−/−*^ mice	The mRNA levels of *Mettl3*, *Mettl14* and *Wtap*, which encode methyltransferases, were significantly reduced in the hippocampus	Learning and memory deficits; Tau, p-Tau, Aβ, soluble A*β*_40_ and A*β*_42_ levels were increased		Lv et al. (2022)
*Mettl14*-KO mice (Conditional deletion of *Mettl14* in striatonigral and striatopallidal neurons)	The total level of m6A was significantly reduced		Associated with synaptic plasticity, learning and memory	Koranda et al. (2018)
*Mettl14* ^* fl/fl*^ *;Olig2*-Cre mutant mice/*Mettl14* ^* fl/fl*^ *;Olig2*- Cre mutant oligodendrocy	METTL14 expression was decreased in the white matter region of the central nervous system		METTL14 plays an important role in postmitotic oligodendrocyte maturation; Central nervous system myelination and maturation are dependent on METTL14	Xu et al. (2020)
*Fto* Tg mice	Expression of PRRC2A was decreased	Displayed significant locomotive and cognitive defects; Demethylation	*Fto* transgene reduced oligodendroglial lineage-specific gene expression; FTO significantly decreased the binding capacity of *Prrc2a* and *Olig2* mRNA and decreased the half-life of *Olig2* mRNA	Wu et al. (2019)
HSV-Cas9-Fto mice			Mediate translation of memory-related transcripts	Walters et al. (2017)
3xTg-AD mice	Expression of FTO was increased	Cognitive deficits; Amyloid plaques and NETs tangles	FTO promote the accumulation of phosphorylated Tau in neurons; FTO activated Tau phosphorylation in a mTOR signaling pathway-dependent manner	Li et al. (2018)
*Prrc2a* ^*f/f*^ *;Nestin* ^*Cre+/−*^ mice/Oligodendrocyte progenitor cells (OPCs)	Expression of PRRC2A was decreased	Hypomyelination, impaired locomotive, and cognitive disability	*Prrc2a* knockout decreased oligodendrocyte gene expression, inhibited the generation and proliferation of OPCs, and resulted in hypomyelination in the brain. Prrc2a mediates myelination by interacting with Olig2 mRNA	Wu et al. (2019)
*Ythdf1*-KO mice	Expression of YTHDF1 was decreased	Learning and memory defects and impaired hippocampal synaptic transmission and long-term potentiation	Promotes the formation of synapses	Shi et al. (2018)
*Ythdf2*-KO zebrafish			It affects the development of early zygotes in mammalian oocytes	Ivanova et al. (2017)
Lps-induced inflammatory mice/microglia cells	IGF2BP1 expression was upregulated in microglia cells	Neuroinflammtion	A positive correlation between the m6A methylation and expression of proinflammatory genes; Igf2bp1 mediates microglial activation via stabilizing Gbp11 and Cp mRNA.	Ding et al. (2022)
iPSC-derived neurons treated with *NSun2* knockdown	Expression of NSun2 was decreased	A significant increase in the levels of phosphorylated tau	NSun2 knockdowwn promoted the level of phosphorylated tau increasing	Kim et al. (2022)
Human A*β*_42_ Tg *Drosophila* melanogaster (fruit flies);*NSun2* KO mice	Expression of NSun2 was decreased	A significant increase in the levels of phosphorylated tau	NSun2 regulates Tau phosphorylation by mediating the methylation level of miR-125b; NSun2 overexpression partially rescues Aβ-induced toxicity	Kim et al. (2022)
Rat primary hippocampal neurons treated with AβO	Expression of NSun2 was decreased	A significant increase in the levels of phosphorylated tau		Kim et al. (2022)
PS19 P301S tau mice/SH-SY5Y, N2a and HEK cells	HNRNPA2B1 expression was upregulated	tau pathology	HNRNPA2B1 promotes Tau oligomerization and is required for the binding of Tau oligomers to m6A; Knockdown of HNRNPA2B1 reduced the tau-induced increase in Caspase 3 expression	Jiang et al. (2021)

### 5.1 Mouse

Mice have been a major tool for AD research because they share most of the major brain regions and neurotransmitter systems with humans. During aging, mouse brains show the same elevated levels of m6A methylation as human brains, as well as a high degree of overlap between m6A sites in mouse brains and human brains (Shafik et al., 2021). A majority of AD mouse models are transgenic models. Edited amyloid precursor protein (APP) is closely associated with AD, and APP is broken down by *ß*-protease and γ-protease to produce toxic Aβ plaques. APP single transgenic mice were first applied to the study of AD based on the hypothesis of insoluble amyloid in the brain of AD patients (Games et al., 1995). APP single transgenic mice exhibited Aβ pathological plaques but did not exhibit neurofibrillary tangles or significant neuronal loss. In preclinical studies for AD drugs, APP transgenic mice are used as preclinical study models to evaluate drugs such as BACE1 and aducanumab monoclonal antibodies, etc (Kastanenka et al., 2016; Neumann et al., 2018).

PS19 transgenic mice express mutant human microtubule-associated protein tau (MART) driven by the mouse prion protein (Prnp) promoter. At 8 months of age, PS19 mice with mutant human tau expression were found to have neuronal loss and brain atrophy in the hippocampus. In other brain regions, extensive neuronal fibrillary tangles were formed, accompanied by microgliosis and astrocytosis (Yoshiyama et al., 2007). RNA transcription is regulated by HNRNPA2B1, a reading protein for m6A in the nucleus. Moreover, HNRNPA2B1, along with other reader proteins, has been shown to promote the interaction between oTau and m6A transcripts in the cytoplasms (Jiang et al., 2021). It was found that HNRNPA2B1 expression increased in the cytoplasm of brain neurons of three-month-old P301S tau mice and was stably co-localized with m6A by 9 months of age. Conversely, HNRNPA2B1 expression was limited to the nucleus of WT mice aged 3–6 months (Jiang et al., 2021). In addition to the classical single transgenic mouse models described above, knockout or overexpression of m6A modifying enzymes in mice also exhibited similar AD phenotypes. The brains of *Mettl3* ^*−/−*^ mice and *Ythdf1* ^*−/−*^ mice showed cognitive impairment similar to AD, in which the brains of *Mettl3* ^*−/−*^ mice showed microglia activation, and METTL3 overexpression mice prevented AβO-induced synaptic loss and cognitive impairment (Shi et al., 2018; Zhao et al., 2021). *Mettl14* ^*−/−*^ mice exhibited white matter abnormalities (Koranda et al., 2018). Similarly, the brains of *Prrc2a* ^*−/−*^ mice also showed hypomyelination and behavioral and cognitive impairments, as did Fto overexpression mice (Wu et al., 2019). The brain of *NSun2* ^*−/−*^mice showed significantly increased levels of phosphorylated Tau (Kim et al., 2022).

In APP/PS1 transgenic mice, pathological changes in the brain are similar to those observed in AD patients, as well as behavioral and cognitive dysfunction. The levels of m6A methylation were elevated in the cortex and hippocampus of APP/PS1 transgenic mice, along with increased expression of METTL3 and decreased expression of FTO (Han et al., 2020). As compared with the APP mouse model, the APP/PS1 mouse brain showed Aβ deposition at an early stage. Neither model was capable of generating NET tangles and complete neuronal loss. Due to this, APP/Tau double transgenic mice develop both Aβ plaque deposition and neurofibrillary tangles. 5×FAD mice exhibit neuronal loss and memory deficits associated with amyloid pathology (Oakley et al., 2006). As an important animal model to ensure clinical marketing of novel AD drugs, the AD transgenic mouse model can predict almost all of the side effects observed in human trials. The triple transgenic 3×Tg-AD mice behaviorally exhibit moderate to severe behavioral impairment of AD, downregulation of FTO expression, formation of Aβ plaques and NETs, as well as gliosis, synaptic damage, and neuronal loss in neuropathology (Wang et al., 2021). TYRO protein kinase-binding protein (TYROBP) knockout mice develop learning and memory deficits as well as increased levels of phosphorylated Tau and Aβ amyloid in the brain. The severity of these symptoms and pathological features increases with age. The deficiency of TYROBP appears to be associated with the regulation of m6A RNA methylation. A decrease in the expression levels of m6A methyltransferases METTL3, METTL14, and WTAP was observed in the brains of *Tyrobp* ^*−/−*^ mice compared to wild type mice, which is consistent with this trend found in the brains of AD patients (Zhao et al., 2021; Lv et al., 2022).

### 5.2 Rat

Rat tau protein more closely resembles human tau protein, and human amyloid precursor protein (APPsw) and progerin 1 (PS1ΔE9) mutant TgF344-AD rats phenotypically exhibit amyloid plaques, age-dependent cognitive deficits, amyloid-β oligomers, glial cell proliferation, and forebrain apoptotic neural loss typical of AD (Cohen et al., 2013). The accumulation of tau protein in the blue spot region and noradrenergic dysfunction may play a critical role in AD disease progression clinically (Braak et al., 2011). Cognitive performance in old age is significantly influenced by the integrity of the nucleus ceruleus (LC)-norepinephrine system (NE) (Mather and Harley, 2016). A preclinical model of this pathological feature may be provided by TgF344-AD rats. TgF344-AD rats have hyperphosphorylation of tau in the locus ceruleus, which is associated with a deficiency in norepinephrine in the hippocampal area (Rorabaugh et al., 2017).

### 5.3 Zebrafish

In terms of biomedical research, zebrafish is an important model vertebrate and is widely used in the study of human disease and development. This species is characterized by a fast reproduction rate, a low cost, and ease of observation. According to genetic analysis, zebrafish and humans share an average genetic similarity of 87%. A zebrafish has the neural structure of vertebrates, and its brain has a similar structure to that of a mammal (Kalueff et al., 2014). A Furthermore, zebrafish have a functional blood-brain barrier (BBB), which does not develop during embryonic life, allowing drugs to enter the brain and may thus be used to screen drugs for neurodegenerative disorders (Kim et al., 2017). Zebrafish have many genes and neural systems that are involved in AD (Santana et al., 2012). In current pharmacological research on brain dysfunction and related drugs, zebrafish, both juvenile and adult, are used (Kalueff et al., 2014). As a translucent vertebrate, zebrafish are physiologically homologous to *Homo sapiens*, making them an ideal model for studying pathways and mechanisms relevant to human disease prognosis and clinical treatment. The AD model with Aβ starch deposition can be constructed by injecting Aβ42 polypeptide into the ventricles of zebrafish. The zebrafish model of AD also exhibits a number of pathological characteristics of the disease, including apoptosis, inflammation, reduced synapses, and cognitive impairment (Bhattarai et al., 2016).

The brain tissue of zebrafish contains a significant amount of m6A modifications (Li et al., 2022). There is evidence that the m6A reader Ythdf in zebrafish is capable of deadenylating maternal mRNA and causing attenuation of maternal zebrafish transcripts, and the sequence of Ythdf in zebrafish is similar to the sequence of Human YTHDFs (Kontur et al., 2020). Similarly, loss of YTHDF2 in mammals affects early zygote development in mammalian oocytes (Ivanova et al., 2017). METTL3 and WTAP knockouts resulted in severe embryonic defects in zebrafish embryos. The knockout of WTAP caused zebrafish to have developmental defects, such as smaller eyes and ventricles, whereas the knockout of Mettl13 had only a minor effect on the embryos. *In situ* hybridization of zebrafish whole tissues revealed that WTAP and METTL3 were commonly expressed early in embryonic development and enriched in brain regions at 36 hpf (Ping et al., 2014). Thus, the development of zebrafish is dependent on m6A methyltransferases. In both rodent and human brains, the methyltransferase family protein METTL5 has been found to be enriched in cellular elements and synapses of hippocampal neurons, and knockdown of the METTL5 gene in zebrafish results in the same phenotype as in humans (Richard et al., 2019).

Zebrafish spinal cord contains different functional OPC subtypes (Marisca et al., 2020). The rapid culture cycle of zebrafish allows the molecular characteristics of oligodendrocyte differentiation at each time period and the changes of m6A modification in oligodendrocyte differentiation by single-cell RNA sequencing. In zebrafish, the heterogeneity of microglia associated with neuroinflammation is almost preserved into adulthood, whereas in other rodents the opposite is true (Li et al., 2019; Silva et al., 2021). The findings of this study will help us establish a zebrafish model of AD to study the role of microglia and m6A modification in the development of AD. The mammalian neural stem/progenitor cells (NSPCs) has a limited ability to regenerate neurons, and the neurons in the brains of people with Alzheimer’s disease cannot grow new neurons once they have lost them (Tanaka and Ferretti, 2009). In contrast to mammals, zebrafish neurons are capable of regeneration (Kyritsis et al., 2012). Thus, the zebrafish model of AD can be designed to study the molecular differences between NSPCs and mammalian NSPCS, and to then utilize them as a form of neuronal regeneration therapy (Cosacak et al., 2015). A variety of new gene editing technologies have recently made it possible to develop zebrafish models of neurodegenerative diseases with greater feasibility and selectivity. A technology for zebrafish that is designed to knock out specific tissues and cell types through a conditional knockout switch method, zCKOIS (zebrafish Conditional Knockout-knock In Switch). By inserting donor plasmids containing reverse knockout markers indicated by red fluorescent protein and forward knockout markers indicated by green fluorescent protein into the gene through Cas9 site-directed operation in the gene intron, it utilizes the efficiency of non-homologous recombination. Combined with tissue-specific Cre expression, it achieves visual gene knockout in specific tissues. Using this method, gene-targeted knockouts in zebrafish can be efficiently performed, which fills a technical gap for studying gene function in zebrafish (Li et al., 2020b).

### 5.4 Organoid

Human-specific pathologies are often not replicated by animal models, and the complex interactions in the *in vivo* environment are not properly reflected by cell model cultures. Organoids are 3D cell cultures that are derived from adult stem cells *in vitro* and have similar histological characteristics to human organs. Organoids can also partially reproduce the physiological functions of human organs. Organoid culture, however, requires a relatively long culture period. In 16–20 days, human fibroblasts can be transformed into specific types of neurons by sequentially adding small molecule inhibitors to the cell culture medium every other day (Hu et al., 2015). A 3D brain organoid can be used as a tool to study brain development, differentiation of brain cells, aging, metabolic processes, drug screening, and disease modeling. Organoids are induced by region-specific growth factors, differentiation factors, and cell inhibitors, which can facilitate the study of the expression level of m6A in different populations of the AD brain, as well as the pathogenic mechanism that causes the disease.

Currently, human pluripotent stem cells (hPSCs) can be used to generate AD organoid models in three different ways. The first method involves the use of Aβ42 oligomers or inducers to induce the generation of AD models (Pavoni et al., 2018). Alternatively, iPSCs can be generated from AD-mutant cells and induced to differentiate into various types of neural cells (Gonzalez et al., 2018). CRISPR/Cas9 gene editing can be used to generate AD organoids by causing mutations in APP, PS1, and APOE4 genes (Lin et al., 2018). Organoids derived from hPSCs have been successfully used to model various neurological disorders, and AD organoids can be created by overexpressing mutant PS1 and APP proteins (Choi et al., 2014). Organoid brain models of AD derived from iPSCs of familial AD patients exhibit progressive accumulation of Aβ protein accompanied by the development of NFTs associated with amyloid plaques (Gonzalez et al., 2018). The construction of AD models using microfluidics by neurons, astrocytes, and microglia can provide an insight into key features of AD pathology, such as Aβ amyloid deposition, tau hyperphosphorylation, neuroinflammation, and neurotoxicity (Park et al., 2018). In addition to being more accurate than other models, the model can be used to develop and screen drugs for interaction with neuroglial cells. Organoids can offer the same advantages as cell cultures as well as the ability to model the complex interactions between cells. It is important to note, however, that studying AD on the basis of AD organoids still have some limitations. As AD is a disease associated with aging, genetic changes as well as changes in the global transcriptional profile of cells accompany the aging process. In spite of this, it is difficult to mimic aging-related phenotypes in iPSCs-induced organoids (Camp et al., 2015; Gerakis and Hetz, 2019). Additionally, AD organoids fail to consider the effects of oxygen and nutrients transported from blood vessels on neuronal cells and their connections.

## 6 Techniques and methods of m6A studies

As the study of m6A modifications has intensified, it has become urgent to develop detection techniques and research methods for m6A modifications. There have been several advances in the detection of m6A modifications in recent years in order to meet the needs of most researchers.

### 6.1 Detection techniques for m6A

As the first developed method, methylated RNA immunoprecipitation (MeRIP) utilizes m6A specific antibodies to recognize methylated RNA on mRNA and RNA immunoprecipitation to enrich methylated fragments (Dominissini et al., 2012). As part of this method, high-throughput sequencing and quantitative PCR were used to analyze RNA regions with m6A methylation modification. However, this method is limited by the lack of specific antibodies or compounds, and by the inability to identify individual modifications of RNA molecules. On the basis of differential cleavage of ribonucleases, the MAZTER-seq system was able to quantify single nucleotides at 16%–25% of the m6A expression sites (Garcia-Campos et al., 2019). Nevertheless, this approach is only able to quantify a subset of m6A modification sites that are located at ACA sites and are situated at an appropriate distance from adjacent ACA sites. The m6Anet is a MIL-based neural network model that identifies potential m6A sites directly from RNA-Seq data and can be applied across multiple cell lines and species without reducing its precision (Hendra et al., 2022). As a result of m6Anet, the stoichiometry of modification is quantified at the site level without the use of control samples, providing further insight into the relationship between RNA modifications and m6A methylation (Hendra et al., 2022). With APEX seq, adjacent RNAs are labeled using the peroxidase enzyme APEX2, which allows for high-resolution RNA mapping of diverse subcellular sites at the nanoscale. It may be applied to any subcellular region, and it allows for the comparison of RNA variants and isoforms (Fazal et al., 2019). The method of CAP-seq involves photooxidation of RNA bases prior to photoactivation, followed by affinity purification and enrichment for sequencing (Wang et al., 2019).

### 6.2 Targeted m6A editing techniques

As of now, the most common method for manipulating RNA methylation modification is to regulate the expression of RNA methyltransferases and demethylases. Techniques to target RNA methylation have been less widely reported. A metabolite of riboflavin kinase, flavin mononucleotide (FMN), mediates substantial demethylation of RNA m6A residues in living cells under blue light irradiation (Xie et al., 2019). However, FMN is unable to modify RNA m6A in a targeted manner. By targeting specific genes, the CRISPR/Cas system is a simple and stable gene editing technology. A mutation in the structural domain of the Cas13 nuclease protein can lead to the generation of a catalytic death enzyme that binds with affinity to RNA (Abudayyeh et al., 2016). Accordingly, recent studies have demonstrated the effectiveness of targeting specific mRNAs for demethylation via CRISPR-Cas13b (Li et al., 2020a). Compared to Cas9, the gRNA of the Cas13 system has no PAM targeting sequence restrictions or motif preference around the target site and has a higher mismatch tolerance (Cox et al., 2017). There is a novel nuclease, MTR1, that can transfer a methyl group to a nitrogen atom of a target RNA, thus adding methyl groups to both synthetic and natural RNA strands (Scheitl et al., 2020). This novel nuclease may be useful in understanding the interplay between RNA methylation, structure, and function. Furthermore, it can be used as an RNA probe to dynamically observe changes in m6A methylation levels over during the course of the disease.

## 7 Summary

This review summarizes the key role of m6A modification in AD, and we additionally summarize the research models of Alzheimer’s disease and the studies related to m6A among them, as well as the current m6A research technologies in recent years. There is an emphasis on the potential role of m6A modification in the development of AD. So far, several RNA m6A modifying enzymes and expression changes of m6A modifying enzymes have been discovered during the development of AD. Nevertheless, many knowledge gaps remain to be filled. The brain environment is complex, and the substances transported by blood vessels, the information transmission between synapses, and the interconnection between cell populations can affect the expression level of key proteins. Proteases involved in the modification of m6A have only recently been discovered. It remains unclear where they are localized in different tissues, what the preferential transcript modification sites are, and what is involved in disease pathogenesis. In AD brain, different modifying enzymes are differentially expressed in different cell populations and involve different signaling pathways. The expression of METTL3 is decreased in the hippocampus of AD brain, leading to cognitive impairment, but its expression is increased in microglia, which has the role of promoting neuroinflammation (Zhao et al., 2021; Wen et al., 2022). The direction of its effect on the pathological development of AD was also opposite in these two regions. Interestingly, METTL3 was highly expressed in insoluble deposits in the brain (Huang et al., 2020). This may be related to the role of Aβ in promoting the activation of microglia, and the specific mechanism needs to be further studied. Through gene targeted knockout, the role of m6A modifying enzymes in different cell populations should be considered to screen out the key factors that can prevent or reverse the disease. Whether m6A modifying enzymes can be used as AD biomarkers in plasma needs further study.

AD presents several diagnostic and therapeutic challenges. First, AD has the characteristic features of long latency and inconspicuous clinical manifestations in its early stages. This is the reason why patients often come to the hospital for tests after the MCI stage. The growing incidence of AD has also placed a significant burden on nursing care. Second, based on the current theoretical conception of AD, it is not yet possible to identify the key pathogenesis of AD and the limitations of current drugs for its treatment. A new perspective on the pathogenesis of AD is needed, as is an exploration of its characteristic biomarkers. In AD brains, m6A methylation levels are abnormal and its modification enzymes have been linked to pathological processes such as synapse loss, oligodendrocyte differentiation, and neuroinflammation. Several new detection methods and research techniques have been reported in recent years to address the limitations of m6A studies. However, research on the pathogenesis of AD at the level of m6A methylation remains shallow. Consequently, this provides a greater opportunity for further analysis of the potential role of m6A methylation modification in AD.
